# Supplementary material for: Niche-Aware Metagenomic Screening for Enzyme Methioninase Illuminates Its Contribution to Metabolic Syntrophy
Source: Microb Ecol. 2024 Nov 15;87(1):141. doi: 10.1007/s00248-024-02458-0 (PMC11568061; doi:10.1007/s00248-024-02458-0)
Supplement: Supplementary file 1 — Supplementary file1 (DOCX 3522 KB) [file 248_2024_2458_MOESM1_ESM.docx]

**Supplementary Figures:**


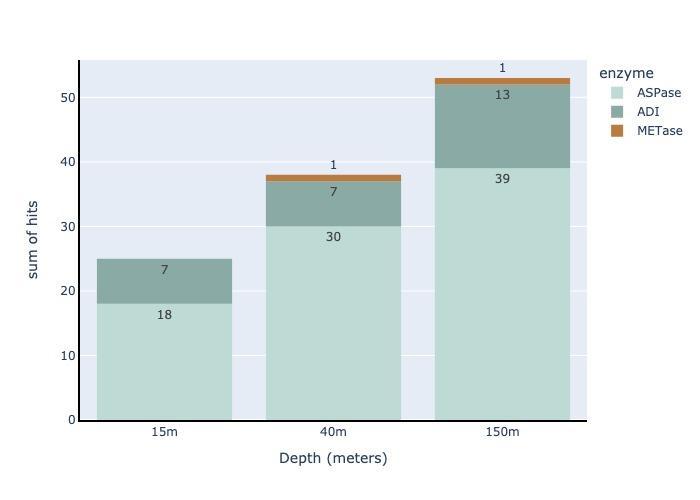


**Supplementary Figure S1-** Distribution of asparaginase, arginine deiminase and methioininase in metagenomic samples collected from different depths of the Caspian Sea brackish water.


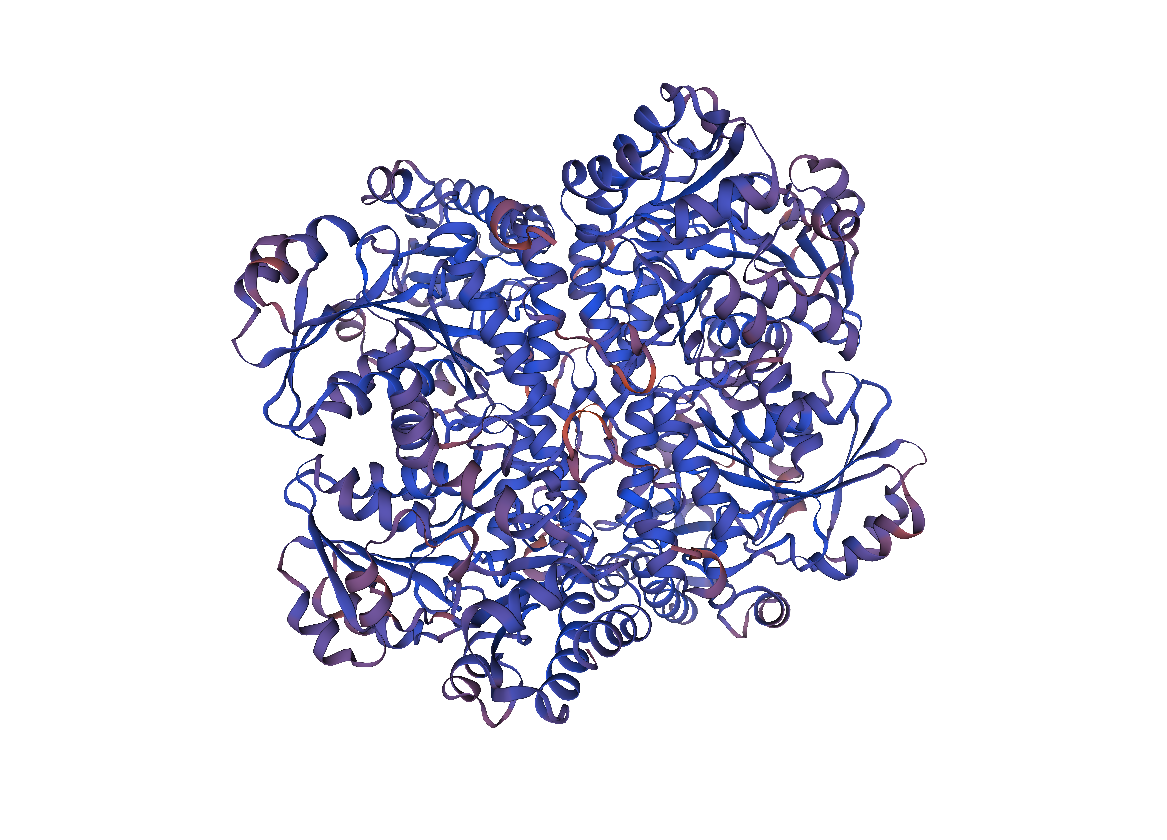

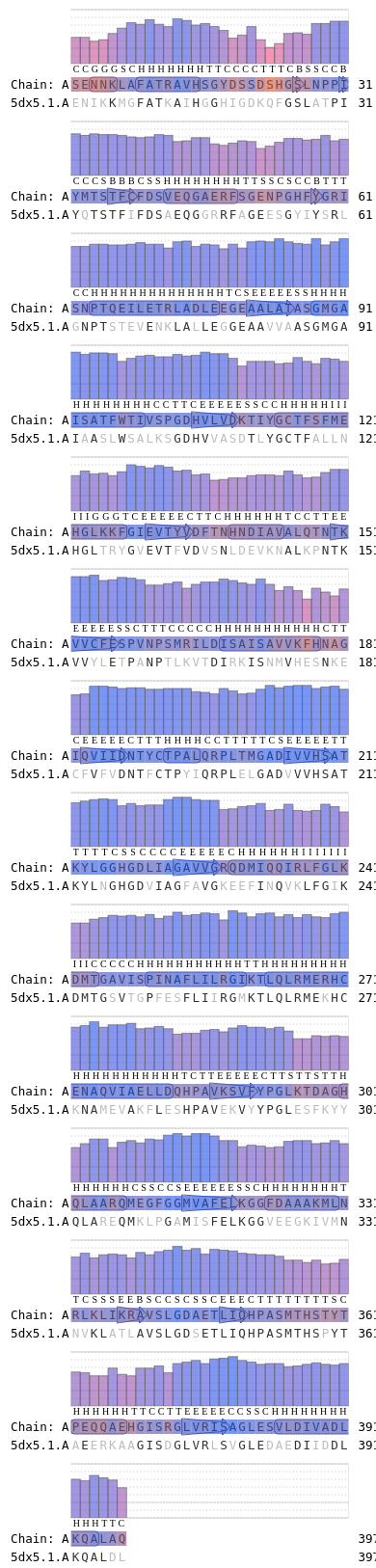

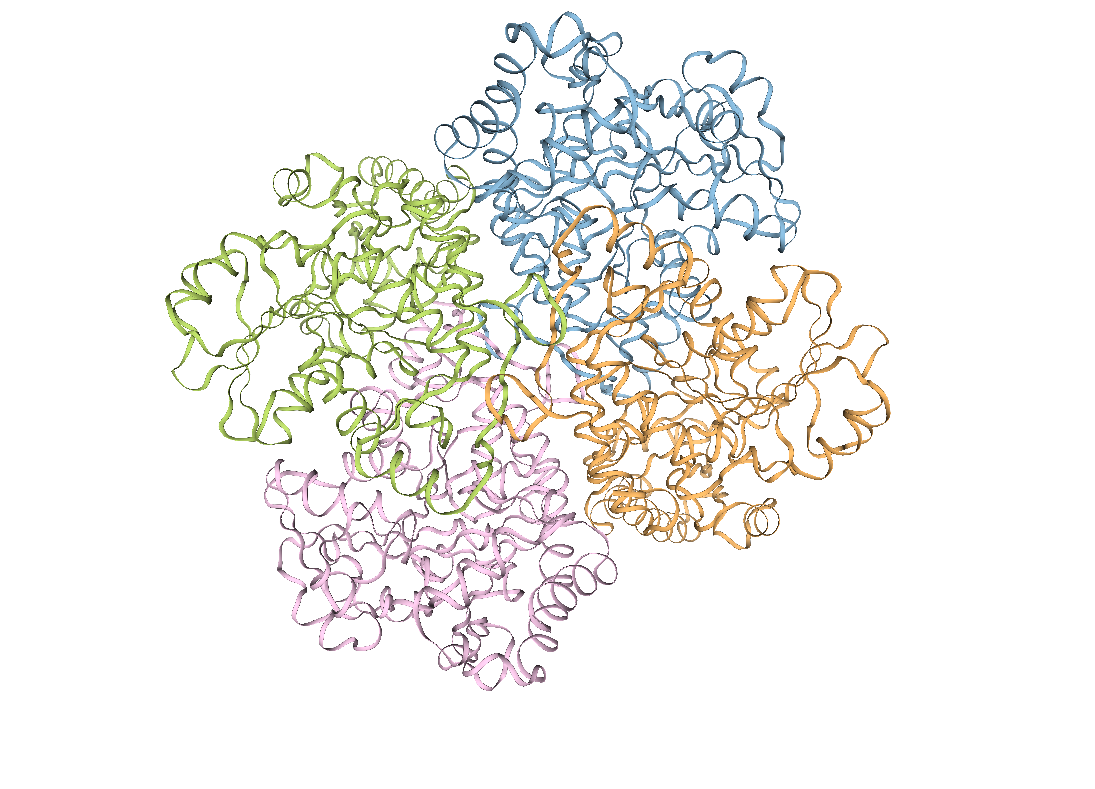


**Supplementary Figure S2-** predicted 3D structure of the methioninase screened from Caspian Sea metagenome. The alignment (left) depicts the high similarity of the screened methioinase protein sequence with the Clostridium sporogenes (strain ATCC 15579) methioninase (5dx5).


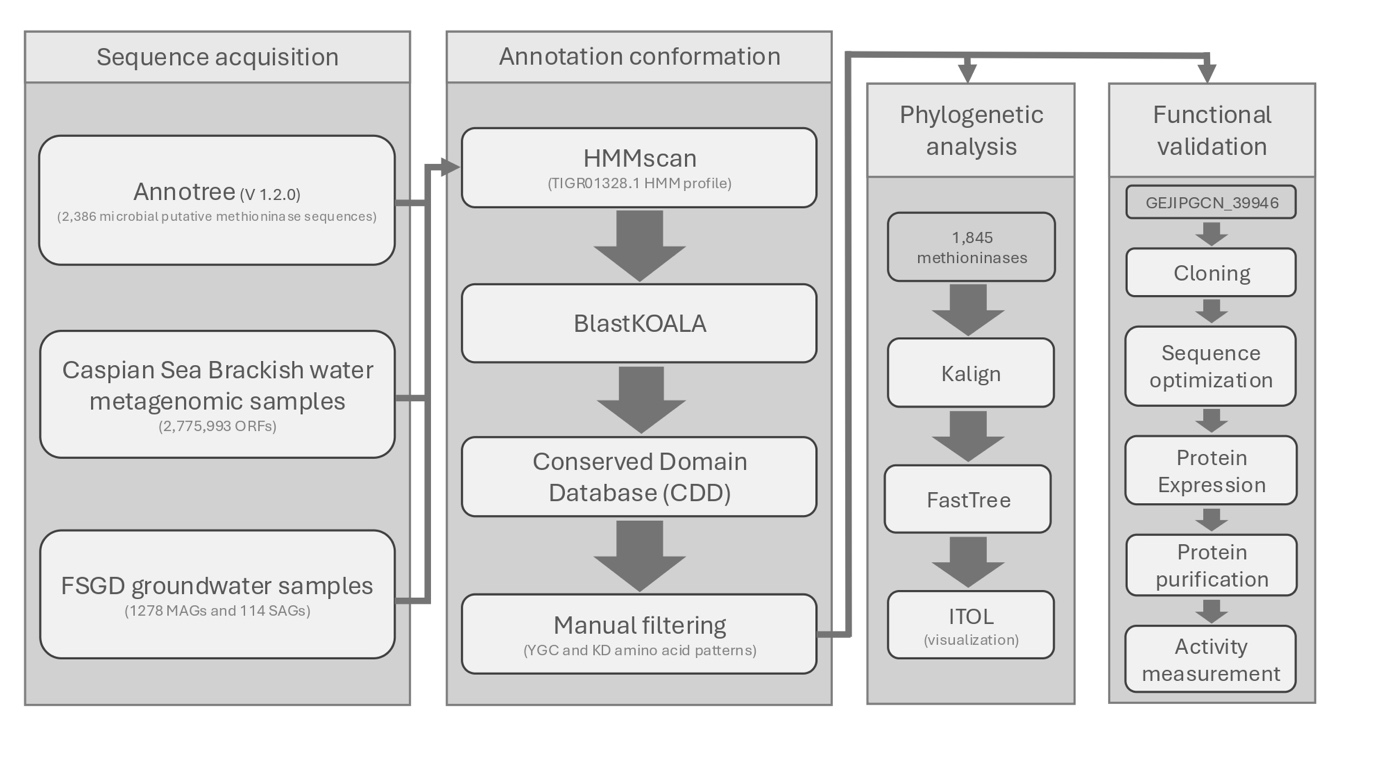


**Supplementary Figure S3-** Stepwise summary of methioninase in silico screening and in vitro verification of the function.
